# Gene characteristics of the complete mitochondrial genomes of *Paratoxodera polyacantha* and *Toxodera hauseri* (Mantodea: Toxoderidae)

**DOI:** 10.7717/peerj.4595

**Published:** 2018-04-19

**Authors:** Le-Ping Zhang, Yin-Yin Cai, Dan-Na Yu, Kenneth B. Storey, Jia-Yong Zhang

**Affiliations:** 1 College of Chemistry and Life Science, Zhejiang Normal University, Zhejiang, China; 2 Key lab of Wildlife Biotechnology, Conservation and Utilization of Zhejiang Province, Zhejiang Normal University, Jinhua, Zhejiang, China; 3 Department of Biology, Carleton University, Ottawa, Ontario, Canada

**Keywords:** Mitochondrial genome, Toxoderidae, Extra trnA and trnR, Intergenic regions, Phylogenetic relationship

## Abstract

The family Toxoderidae (Mantodea) contains an ecologically diverse group of praying mantis species that have in common greatly elongated bodies. In this study, we sequenced and compared the complete mitochondrial genomes of two Toxoderidae species, *Paratoxodera polyacantha* and *Toxodera hauseri*, and compared their mitochondrial genome characteristics with another member of the Toxoderidae, *Stenotoxodera porioni* (KY689118)*.* The lengths of the mitogenomes of *T. hauseri* and *P. polyacantha* were 15,616 bp and 15,999 bp, respectively, which is similar to that of *S. porioni* (15,846 bp). The size of each gene as well as the A+T-rich region and the A+T content of the whole genome were also very similar among the three species as were the protein-coding genes, the A+T content and the codon usages. The mitogenome of *T. hauseri* had the typical 22 tRNAs, whereas that of *P. polyacantha* had 26 tRNAs including an extra two copies of *trnA*-*trnR*. Intergenic regions of 67 bp and 76 bp were found in *T. hauseri* and *P. polyacantha*, respectively, between *COX2* and *trnK*; these can be explained as residues of a tandem duplication/random loss of *trnK* and* trnD.* This non-coding region may be synapomorphic for Toxoderidae. In BI and ML analyses, the monophyly of Toxoderidae was supported and *P. polyacantha* was the sister clade to* T. hauseri* and* S. porioni*.

## Introduction

Mantodea are a major group of predatory insects and over 2,500 extant species/subspecies are known that belong to 427 genera, assigned to 21 families (Ehrmann, 2002; Svenson & Whiting, 2009; Otte, Spearman & Stiewe, 2016). Toxoderidae (Mantodea) was originally listed as a subfamily of Mantidae (Beier, 1935) but Ehrmann (2002) revised its status to the family rank. In Svenson & Whiting’s research (2009), three Toxoderidae species (*Aethalochroa* sp., *Toxoderopsis taurus* and *Stenotoxodera porioni*) formed a monophyletic group which was the sister clade to Oxyothespinae (Mantidae). Zhang et al. (2018a) found that *Stenotoxodera porioni* and *Schizocephala bicornis* (Mantidae) were a sister group. Praying mantises in this family are ecologically diverse and are distributed across the Indian subcontinent, Indonesia, southwest Asia, tropical Africa, Afghanistan, and Australia (Patel, Sing & Singh, 2016). The outstanding feature of Toxoderidae is a highly elongated body; in particular, the prothorax is very long, often nearly half of the entire body length and the metazona is laterally compressed and often carries a dorsal ridge (Wieland, 2013).

Mitochondrial genomes have been used extensively as molecular markers for phylogenetic analyses and comparative or evolutionary genomic research due to their features that include small genome size, fast evolution rates, low sequence recombination, and evolutionary conserved gene products (Boore, 2006; Zhang et al., 2008; Cameron, 2014a; Ma et al., 2015; Cheng et al., 2016). The typical insect mitogenome is a 14–20 kb circular molecule including 37 genes (13 protein-coding genes, two ribosomal RNA genes, and 22 transfer RNA genes) and an A+T-rich region, all on a single chromosome (Boore, 1999; Cameron, 2014a). In insect mitochondrial genomes, gene rearrangements are frequently observed (Oliveira et al., 2008; Beckenbach & Joy, 2009; Dowton et al., 2009; Leavitt et al., 2013; Wei et al., 2014; Dickey et al., 2015), but gene duplications (extra copies) or deletions (gene loss) are rarer events (Cameron, 2014a). In terms of gene duplication, many species show an extra tRNA gene copy near the A+T-rich region, supporting the idea that gene duplication events are mainly due to replication slippage mechanisms (Macey et al., 1997; Zhang & Hewitt, 1997). For example, an extra copy of *trnM* was found in *Parafronurus youi* (Ephemeroptera) (Zhang et al., 2008) and *Abispa ephippium* (Hymenoptera) (Cameron et al., 2008). A complete duplication of *trnI* occurred in the mitogenomes of *Chrysomya* species (Diptera) (Junqueira et al., 2004; Nelson et al., 2012), *Reduvius tenebrosus* (Hemiptera) (Jiang et al., 2016), *Nasutitermes corniger* (Blattodea) (Dietrich & Brune, 2016) and *Acraea issoria* (Lepidoptera) (Hu et al., 2010). However, an extra tRNA gene copy is also sometimes found in other regions. For example, a duplicated *trnL* (UUR) was identified in *Troglophilus neglectus* (Orthoptera) (Fenn et al., 2008) and an extra copy of *trnR* occurred in *Brontostoma colossus* (Heteroptera) (Kocher et al., 2014). However, the phenomenon of an insect mitochondrial genome with multi-copies of a specific tRNA gene is quite rare. To our knowledge, among published genomes, *Trialeurodes vaporariorum* (Heteroptera) had five copies of *trnS* (UCN) with an identical anticodon in a direct repeat (Thao & Baumann, 2004) and *Apispa ephippium* (Hymenoptera) had four identical copies of *trnL* (UUR) (Cameron et al., 2008). By contrast, among the published complete mitogenomes of mantises, a considerable number of gene rearrangements occur. A survey of the complete mitogenomes of 43 mantis species belonging to nine families (Hymenopodidae, Iridopterygidae, Liturgusidae, Mantidae, Sibyllidae, Tarachodidae, Thespidae and Toxoderidae) (Cameron, Barker & Whiting, 2006; Wang et al., 2016; Ye et al., 2016; Tian et al., 2017; Zhang & Ye, 2017; Zhang et al., 2018a; Zhang et al., 2018b), revealed that three Liturgusidae species (*Humbertiella nada*, *Theopompa* sp.-HN, *Theopompa* sp.-YN) possessed a derived gene arrangement of *trnM*-*trnI*-*trnQ* (Ye et al., 2016). Furthermore, six Hymenopodidae species *Ambivia undata*, *Creobroter gemmata*, *Creobroter jiangxiensis*, *Creobroter urbanus*, *Odontomantis* sp. and *Theopropus elegans*, three Mantidae species *Mantis religiosa*, *Phyllothelys* sp. and *Statilia* sp., and Liturgusidae species *Theopompa* sp.-HN contained two to eight identical *trnR* genes, and *Statilia* sp. also had five copies of *trnW* pseudogenes (Ye et al., 2016; Zhang et al., 2018a). In addition, *Schizocephala bicornis* (Mantidae) had five identical *trnI* and *Stenotoxodera porioni* (Toxoderidae) had three identical *trnK* (Zhang et al., 2018a).

In this study, we sequenced and annotated two complete mitochondrial genomes of Toxoderidae species, *Paratoxodera polyacantha* and *Toxodera hauseri*, and compared them with the mitogenome of another known Toxoderidae species *Stenotoxodera porioni* (Zhang et al., 2018a). Our results supplement and enhance the limited molecular data available for praying mantis species and may give us a useful model for studying the characteristics and mechanisms of tRNA duplications.

## Materials and Methods

### Sampling collection and DNA extraction

Two samples *P. polyacantha* and *T. hauseri* were collected from Borneo island in 2015, identified by JY Zhang and stored in 100% ethanol at −40 °C. Total DNA was extracted from muscle of one leg using the QIAGEN DNeasy Blood and Tissue Kit (QIAGEN, Germany).

### PCR amplification and sequencing

Two mantis mitogenomes were amplified with six pairs of mantis-specific universal primer sets F2, F3, F7, F9, F10 and F11 as described in Zhang et al. (2018a) and specific primers were designed based on the sequenced PCR information from universal primers using Primer Premier 5.0 (Table 1). We used both normal PCR (product length < 3,000 bp) and Long-PCR (product length >3,000 bp) methods with *Takara Taq* and *Takara LATaq* DNA polymerase, respectively (Takara, Dalian, China) in a 50 µL reaction volume. The reaction systems and cycling conditions for normal PCR and Long-PCR were as in Zhang et al. (2018a). All PCR products were sequenced in both directions using the primer-walking method and ABI3730XL by Sangon Biotech Company (Shanghai, China).

**Table 1:** Specific primers used to amplify the mitogenomes of *P. polyacantha* and *T. hauseri*.

Species	Primer name	Sequence (5′–3′)	Product length (bp)
*P. polyacantha*	64-WZ-J-2431	ATCCCATCCTCTATCAACATC	1,600
	64-WZ-N-4047	AGACCATTACTTGCTTTTCAG	
	64-WZ-J-4067	CTGAAAAGCAAGTAATGGTCT	4,900
	64-WZ-N-8996	AGATTAGTAGGGGGATTTTTAG	
	64-WZ-J-4821	GGTACATTATCAATTCGTTT	3,200
	64-WZ-N-8008	GGTTCATTTTTTTTAGTTTT	
	64-WZ-9330	AATAATGGTAAAGAAGCGAAT	4,000
	64-WZ-N-13569	TTTTTGCTCGCCTGTTTAT	
	64-WZ-J-14354	CGATACACCTACTTTGTTACGA	4,000
	64-WZ-N-2567	ACAAATCCCAGAAATCCAATAG	
	64-WZ-J-11521	ATTTCCTATTCGCCTATGC	700
	64-WZ-N-12258	GGTTTGTTTCTTGTCTTGCT	
*T. hauseri*	66-HSJT-J-1799	CACTCTATTTTGTCTTCGG	700
	66-HSJT-N-2511	TTCTTTTTTTCCTCTTTCA	
	66-HSJT-J-3237	ACTTACCTCCCGCTGAA	1,500
	66-HSJT-N-4715	GGAACAAGATGGGCAAA	
	66-HSJT-J-7393	AAAACGAATGTCCTGAA	1,100
	66-HSJT-N-8472	GATTGCCTTTGAACTTG	
	66-HSJT-J-9121	TAAGACACCAGCCAAGA	4,200
	66-HSJT-N-13343	TAAGGGACGAGAAGACC	
	66-HSJT-J-14386	AATAATGAGAGTGACGGGC	2,600
	66-HSJT-N-1337	AAGGAGGATAGAACTAAGATGA	
	66-HSJT-J-15004	TAAAATCATCTACTGCCGA	1,200
	66-HSJT-N-621	CAAAGGAATGAAGGAGAGT	

### Mitogenome annotation and sequence analyses

Contiguous sequence fragments were assembled using DNASTAR Package v.6.0 (Burland, 2000). The tRNA genes and their potential cloverleaf structures were identified by MITOS (http://mitos.bioinf.uni-leipzig.de/index.py) (Bernt et al., 2013) using the invertebrate mitogenome genetic code. Two rRNA genes (12S and 16S rRNA) were determined by comparison with homologous sequences of mtDNA from other mantis species using Clustal X (Thompson et al., 1997). Following identification of tRNAs and rRNAs, 13 protein-coding genes were translated with the invertebrate mitogenome genetic code to find the open reading frames between tRNAs (Cameron, 2014b). We used CG View server V 1.0 (Grant & Stothard, 2008) to draw the mitochondrial genome map with GC content and GC skew of *P. polyacantha* and *T. hauseri.* The A+T content, codon usage and relative synonymous codon usage (RSCU) of protein-coding genes were calculated by Mega 7.0 (Kumar, Stecher & Tamura, 2016). Composition skewness was calculated according to the following formulas: AT-skew = (A − T)/(A + T); GC-skew = (G − C)/(G + C) (Perna & Kocher, 1995).

### Phylogenetic analyses

In order to discuss the phylogenetic relationships of Toxoderidae, 43 previously sequenced mantis mitogenomes (Cameron, Barker & Whiting, 2006; Wang et al., 2016; Ye et al., 2016; Tian et al., 2017; Zhang & Ye, 2017; Zhang et al., 2018a; Zhang et al., 2018b) were used in the phylogenetic analyses. The outgroup taxa were two cockroaches, *Cryptocercus kyebangensis* and *Eupolyphaga sinensis* (Zhang et al., 2010) and two termites, *Termes hospes* (Dietrich & Brune, 2016) and *Macrotermes barneyi* (Wei et al., 2012). Accession numbers of all mitogenomes are listed in the phylogenetic trees. According to the phylogenetic analyses of Zhang et al. (2018a), we used the nucleotide sequences of the 13 protein-coding genes as the dataset to construct the BI and ML phylogenetic trees. Each of 13 protein-coding genes were aligned using Clustal W in the program Mega 7.0 (Kumar, Stecher & Tamura, 2016) and conserved regions were identified by the program Gblock 0.91b (Castresana, 2000). The resulting alignments were concatenated with Geneious 8.1.6 (Kearse et al., 2012). We used the program PartitionFinder 1.1.1 (Lanfear et al., 2012) to infer the optimal partitioning strategy and choose the best model according to the Bayesian Information Criterion (BIC). The data blocks were defined by each of three codon positions for the thirteen protein-coding genes and a total of 11 partitions were found. ML analysis was implemented in RAxML 8.2.0 with a GTRGAMMA model and branch support for each node was evaluated with 1,000 replicates (Stamatakis, 2014). BI analysis was implemented in MrBayes 3.2 with a GTR + I + G model, each of four chains (three hot and one cold), with run length of 10 million generations and sampling every 1,000 generations (Ronquist et al., 2012). Convergence was assessed with Tracer 1.5 (Rambaut & Drummond, 2007) and trees from the first 25% of the samples were removed as burn-in.

## Results and Discussion

### Mitogenome organization and composition

**Figure 1:**
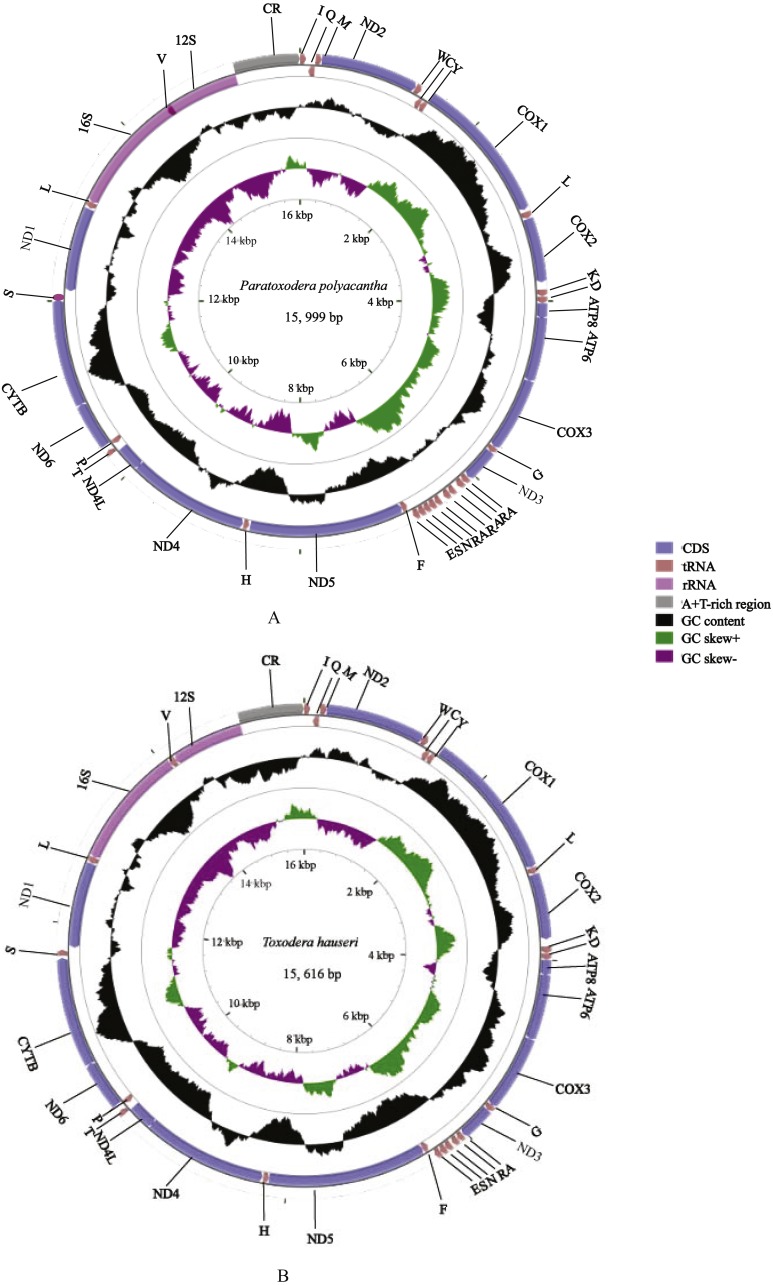
Mitochondrial genome maps of *P. polyacantha* (A) and *T. hauseri* (B). The first circle shows the gene map (PCGs, rRNAs, tRNAs and the AT-rich region) and the genes outside the map are coded on the majority strand (J-strand) whereas the genes inside the map are coded on the minority strand (N-strand). The second circle shows the GC content and the third shows the GC skew. GC content and GC skew are plotted as the deviation from the average value of the entire sequence.

We annotated and deposited the complete mitogenomes of *P. polyacantha* (MG049920) and *T. hauseri* (KX434837) in the GenBank database. There two new mitogenomes were double circular DNA molecules with lengths of 15,999 bp and 15,616 bp, respectively (Figs. 1A–1B). These were longer and shorter, respectively, than the mitogenome of *S. porioni* (15,846 bp), a previously sequenced member of Toxoderidae that we used as a comparison species. The size variation of the three mitochondrial genomes was mainly caused by different intergenic nucleotides (IGNs) and the presence of additional copies of tRNAs in *P. polyacantha* and *S. porioni*. Of all 43 sequenced mantis mitogenomes (Cameron, Barker & Whiting, 2006; Wang et al., 2016; Ye et al., 2016; Tian et al., 2017; Zhang & Ye, 2017; Zhang et al., 2018a; Zhang et al., 2018b), the length of the mitogenome of *Hierodula patellifera* (16,999 bp) was the longest whereas that of *Tenodera sinensis* (15,531 bp) was the shortest. The mitogenome lengths of seven Paramantini species were long (>16,000 bp) because of a large non-coding region (400–1,500 bp) between *trnM* and *ND2* apart from the typical A+T-rich region. The mitogenomes of *Anaxarcha zhengi, Deroplatys desiccate*, *Mantidae* sp., *Parablepharis kuhlii asiatica*, *Phyllothelys* sp1., *Theopompa* sp.-YN and *Theopompa* sp.-YN were also longer than 16,000 bp because of a long typical A+T-rich region (>1,100 bp) (Ye et al., 2016). The mitogenome of *T. hauseri* contained the typical 37 genes (13 PCGs, 22 tRNAs and 2 rRNAs) and an A+T-rich region (Table S1) whereas the mitogenome of *P. polyacantha* had an extra four tRNAs (2 *trnA* and 2 *trnR*) (Table S2); by comparison, *S. porioni* had an extra two *trnK*. The *T. hauseri* mitogenome contained the shortest IGNs, a total of 210 bp, compared to 305 bp for *P. polyacantha* and 313 bp for *S. porioni*. The nucleotide composition of the *P. polyacantha* and *T. hauseri* mitogenomes had a high A+T bias of 74.81% and 73.49% and both showed positive AT-skew and negative GC-skew, which was also similar to *S. porioni* (Table 2).

**Table 2:** Base composition of mantis mitochondrial genomes.

Species name	A+T(%)				AT-skew					GC-skew				
	Mito	PCGs	rRNAs	A+T-rich region	Mito	PCGs-H	PCGs-L	rRNAs	A+T-rich region	Mito	PCGs-H	PCGs-L	rRNAs	A+T-rich region
*P. polyacantha*	74.81	74.43	77.39	76.48	0.044	−0.075	−0.225	−0.039	0.035	−0.195	−0.150	0.263	0.356	−0.210
*T. hauseri*	73.49	73.09	76.39	76.50	0.061	−0.060	−0.247	−0.075	0.023	−0.231	−0.188	0.276	0.418	−0.193
*S.porioni* (KY689118)	73.49	73.00	76.34	76.93	0.058	−0.076	−0.252	−0.079	−0.002	−0.212	−0.174	0.277	0.369	−0.241

### Protein-coding genes and codon usages

All 13 protein-coding genes (PCGs) were identified in the mitogenomes of *P. polyacantha* and *T. hauseri*. Nine PCGs (*ND2*, *COX1*, *COX2*, *ATP8*, *ATP6*, *COX3*, *ND3*, *ND6* and *CYTB*) were coded on the majority strand (J-strand) and the remaining four (*ND5*, *ND4*, *ND4L*, and *ND1*) were coded on the minority strand (N-strand). The length, codon usages and A+T content of PCGs in the *P. polyacantha* and *T. hauseri* mitogenomes were nearly identical to *S. porioni*. Among three mitogenomes, 12 PCGs used ATN (N represents A, T, C, G) as initiation codons with the exception of *COX1* which was initiated with TTG. TTG is an accepted conventional initiation codon for many insect mitogenomes including among mantises (Ye et al., 2016; Zhang & Ye, 2017; Zhang et al., 2018a) and cockroaches (Jeon & Park, 2015; Cheng et al., 2016). TAA was commonly used as for the termination codons although the incomplete termination codon T was found in *COX3* and *ND5* in all three mitogenomes. An incomplete termination codon has also been found in all other sequenced mantis species (Cameron, Barker & Whiting, 2006; Wang et al., 2016; Ye et al., 2016; Tian et al., 2017; Zhang & Ye, 2017; Zhang et al., 2018a; Zhang et al., 2018b). It has been demonstrated that incomplete termination codons can act as functional termination codons in polycistronic transcription cleavage and polyadenylation processes (Ojala, Montoya & Attardi, 1981; Du et al., 2016). In the *P. polyacantha* mitogenome, *COX2* used TAG as the termination codon. Although TAG is the canonical termination codon in insect mitogenomes, it is not used frequently perhaps due to the high percentage of AT nucleotide use by the protein-coding genes (Liu et al., 2016). In the 43 published mantis mitogenomes, only *COX1* of *Theopompa* sp.-YN (Ye et al., 2016) and *Leptomantella albella* (Wang et al., 2016), *COX2* of *Theopropus elegans*, *ATP8* of *Sibylla pretiosa*, *ATP6* of *Phyllothelys* spp. and *Creobroter jiangxiensis*, *ND4* of *Schizocephala bicornis* and *CYTB* of *Creobroter urbanus* (Zhang et al., 2018a) as well as *ND3* of *Tamolanica tamolana* (Cameron, Barker & Whiting, 2006) used TAG as the termination codon.

**Figure 2:**
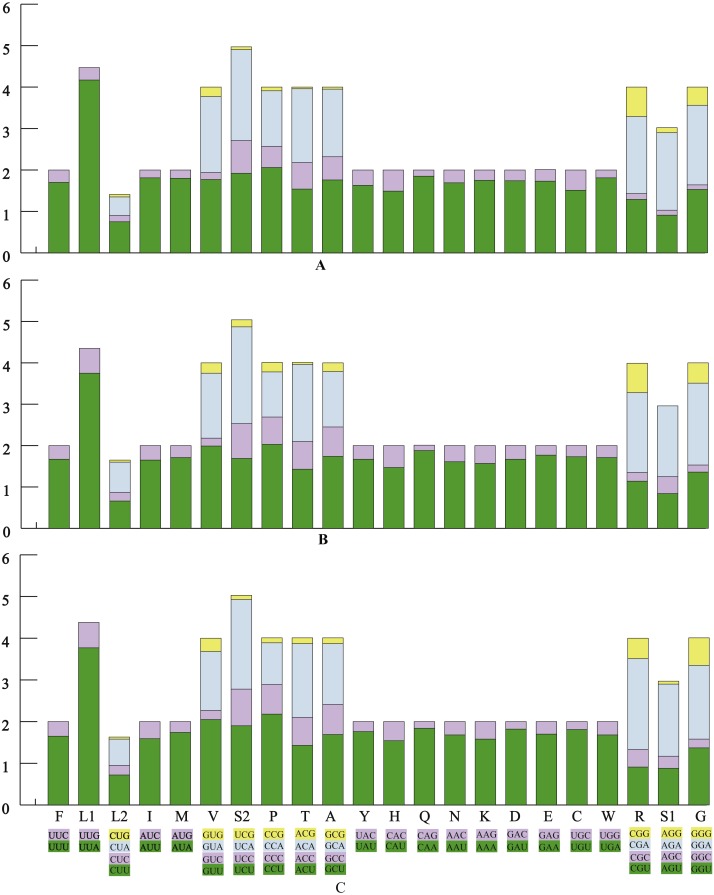
The relative synonymous codon usage (RSCU) in three mantis mitogenomes. The RSCU of the mitogenome in *P. polyacantha* (A), *T. hauseri* (B), and *S. porioni* (C).

**Figure 3:**
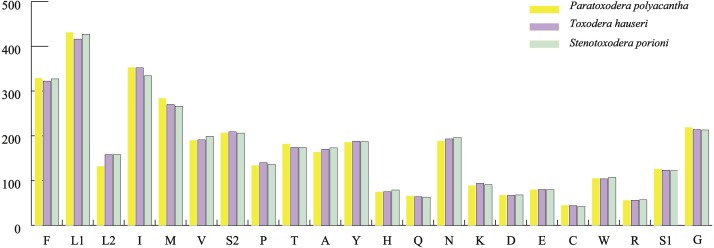
Total codons in three mantis mitogenomes.

The average AT contents of the 13 PCGs in *P. polyacantha* and *T. hauseri* were 74.43% and 73.09%, both slightly higher than *S. porioni* (73%). The PCGs encoded by the majority strand displayed T-skews (the content of T > A) and G-skews (G > C) whereas the minority strand displayed T-skews and C-skews (C > G). We calculated the relative synonymous codon usage (RSCU) of the three Toxoderidae species mitogenomes (Figs. 2A–2C; Table 3) and the result showed that NNU and NNA were higher than 1.0 with the exception of Leu (CUR) and Ser (AGU) in *P. polyacantha*, *T. hauseri* and *S. porioni* and Arg (CGU) only in *S. porioni*. The most frequent amino acids in the coding sequences of *P. polyacantha*, *T. hauseri* and *S. porioni* mitochondrial proteins were Leu (UUR), Ile and Phe (>300) (Fig. 3). These three amino acids were also frequently used in 43 other mantis mitogenomes (Cameron, Barker & Whiting, 2006; Wang et al., 2016; Ye et al., 2016; Tian et al., 2017; Zhang & Ye, 2017; Zhang et al., 2018a; Zhang et al., 2018b) and Lepidoptera mitogenomes (Liu et al., 2016; Xin et al., 2018; Wu et al., 2012). The least used amino acid in the three mitogenomes was Cys (<50).

**Table 3:** The codon number and relative synonymous codon usage in mitochondrial protein coding genes.

Codon	Count			RSCU			Codon	Count			RSCU			Codon	Count			RSCU		
	PP	TH	SP	PP	TH	SP		PP	TH	SP	PP	TH	SP		PP	TH	SP	PP	TH	SP
UUU(F)	279	269	269	1.70	1.67	1.65	UCU(S)	80	70	78	1.92	1.69	1.9	UAU(Y)	152	157	165	1.63	1.67	1.76
UUC(F)	50	53	58	0.30	0.33	0.35	UCC(S)	33	35	36	0.79	0.84	0.88	UAC(Y)	34	31	22	0.37	0.33	0.24
UUA(L)	391	359	368	4.17	3.75	3.77	UCA(S)	91	97	88	2.19	2.34	2.15	CAU(H)	56	55	61	1.49	1.47	1.54
UUG(L)	40	57	59	0.43	0.6	0.61	UCG(S)	3	7	4	0.07	0.17	0.1	CAC(H)	19	20	18	0.51	0.53	0.46
CUU(L)	70	63	70	0.75	0.66	0.72	CCU(P)	69	71	74	2.06	2.03	2.18	CAA(Q)	61	60	58	1.85	1.88	1.84
CUC(L)	14	20	22	0.15	0.21	0.23	CCC(P)	17	23	24	0.51	0.66	0.71	CAG(Q)	5	4	5	0.15	0.13	0.16
CUA(L)	42	70	61	0.45	0.73	0.63	CCA(P)	45	38	34	1.34	1.09	1	AAU(N)	160	155	165	1.69	1.61	1.68
CUG(L)	6	5	5	0.06	0.05	0.05	CCG(P)	3	8	4	0.09	0.23	0.12	AAC(N)	29	38	31	0.31	0.39	0.32
AUU(I)	319	291	266	1.81	1.65	1.59	ACU(T)	70	62	62	1.54	1.43	1.43	AAA(K)	78	74	72	1.75	1.57	1.58
AUC(I)	34	61	68	0.19	0.35	0.41	ACC(T)	29	29	29	0.64	0.67	0.67	AAG(K)	11	20	19	0.25	0.43	0.42
AUA(M)	256	231	231	1.80	1.71	1.74	ACA(T)	81	81	77	1.78	1.86	1.77	GAU(D)	59	56	62	1.74	1.67	1.82
AUG(M)	28	39	35	0.20	0.29	0.26	ACG(T)	2	2	6	0.04	0.05	0.14	GAC(D)	9	11	6	0.26	0.33	0.18
GUU(V)	84	95	102	1.77	1.99	2.05	GCU(A)	72	74	73	1.76	1.74	1.69	GAA(E)	69	71	68	1.73	1.77	1.7
GUC(V)	8	9	11	0.17	0.19	0.22	GCC(A)	23	30	31	0.56	0.71	0.72	GAG(E)	11	9	12	0.28	0.23	0.3
GUA(V)	87	75	70	1.83	1.57	1.41	GCA(A)	67	57	63	1.63	1.34	1.46	UGU(C)	34	38	38	1.51	1.73	1.81
GUG(V)	11	12	16	0.23	0.25	0.32	GCG(A)	2	9	6	0.05	0.21	0.14	UGC(C)	11	6	4	0.49	0.27	0.19
GGU(G)	84	73	73	1.53	1.36	1.37	CGU(R)	18	16	13	1.29	1.14	0.91	AGU(S)	38	35	36	0.91	0.84	0.88
GGC(G)	6	9	11	0.11	0.17	0.21	CGC(R)	2	3	6	0.14	0.21	0.42	AGC(S)	5	17	12	0.12	0.41	0.29
GGA(G)	105	106	94	1.92	1.98	1.77	CGA(R)	26	27	31	1.86	1.93	2.18	AGA(S)	78	71	71	1.87	1.71	1.73
GGG(G)	24	26	35	0.44	0.49	0.66	CGG(R)	10	10	7	0.71	0.71	0.49	AGG(S)	5	0	3	0.12	0	0.07
UGA(W)	95	89	89	1.81	1.71	1.68	UGG(W)	10	15	17	0.19	0.29	0.32							

**Notes.**

PP *P. polyacantha* TH *T. hauseri* SP *S. porioni* (KY689118)

### Ribosomal RNAs and transfer RNAs

The mitogenomes of *P. polyacantha* and *T. hauseri* each had one *16S rRNA* and one *12S rRNA* gene. The *16S rRNA* gene was located between *trnL* (UUR) and *trnV* and the *12S rRNA* gene was located between *trnV* and the A+T-rich region as also occurs in other mantises. The size of the *16S rRNA* was 1,387 bp in *P. polyacantha* and 1,322 bp in *T. hauseri*, both a little longer than in *S. porioni* (1,317 bp). The size of the 12*S rRNA* was 787 bp in *T. hauseri* similar to *S. porioni* (788 bp) whereas the *P. polyacantha* (842 bp) was much longer. In the *P. polyacantha* mitogenome, the A+T content of the rRNA genes was the highest (77.39%) whereas the A+T content of rRNAs in the *T. hauseri* mitogenome was approximately 76%, slightly lower than the A+T-rich region. In *P. polyacantha* and *T. hauseri*, the AT-skew was slightly negative whereas the GC-skew was strongly positive indicating that the contents of T and G were higher than those of A and C, respectively.

**Figure 4:**
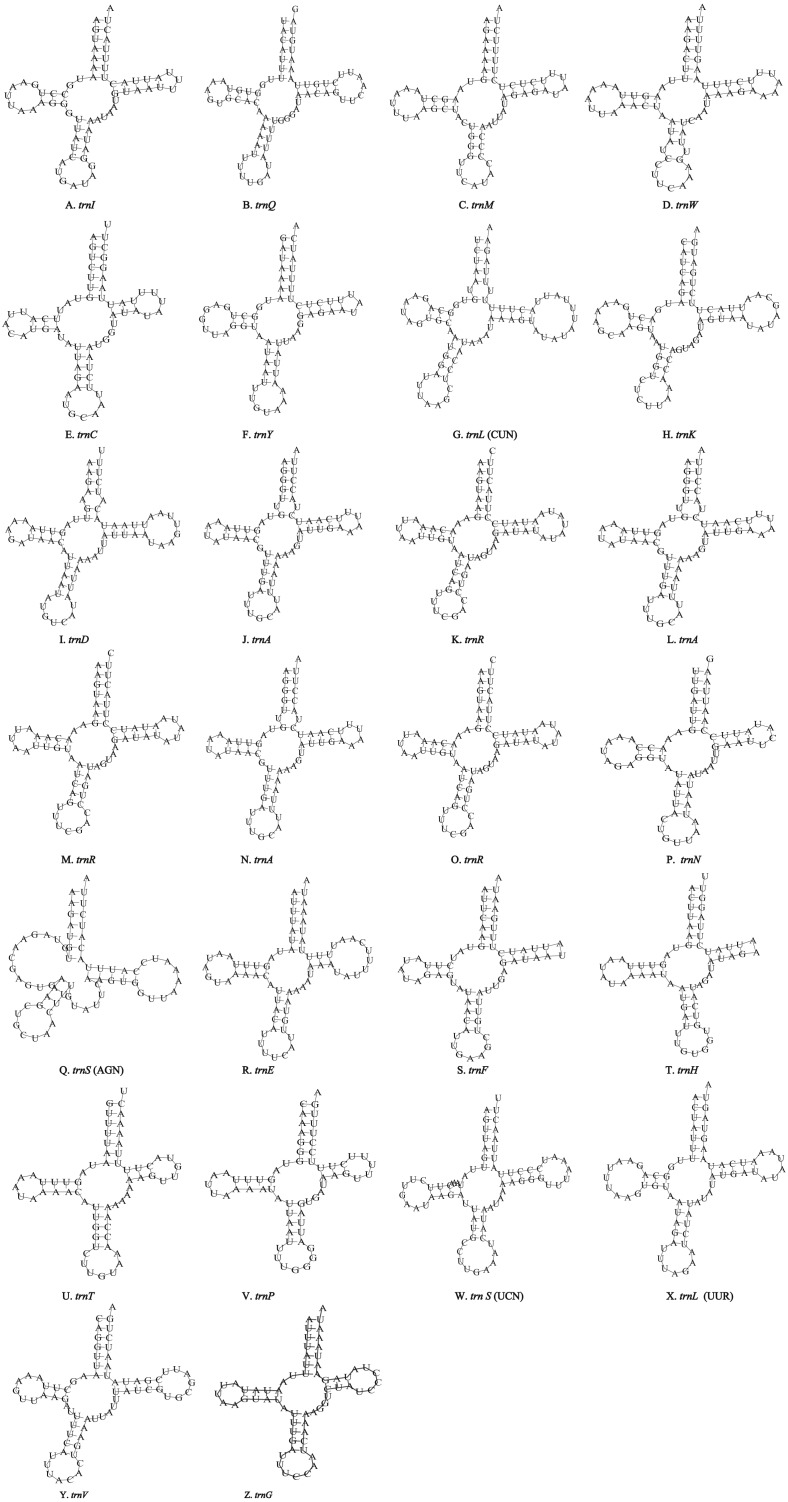
Inferred secondary structures of the 26 tRNA genes of *P. polyacantha* mitogenome. (A) *trnI*; (B) *trnQ*; (C) *trnM*; (D) *trnW*; (E) *trnC*; (F) *trnY*; (G) *trnL* (CUN); (H) *trnK*; (I) *trnD*; (J) *trnA*; (K) *trnR*; (L) *trnA*; (M) *trnR*; (N) *trnA*; (O) *trnR*; (P) *trnN*; (Q) *trnS* (AGN); (R) *trnE*; (S) *trnF*; (T) *trnH*; (U) *trnT*; (V) *trnP*; (W) *trnS* (UCN); (X) *trnL* (UUR); (Y) *trnV*; (Z) *trnF*.

**Figure 5:**
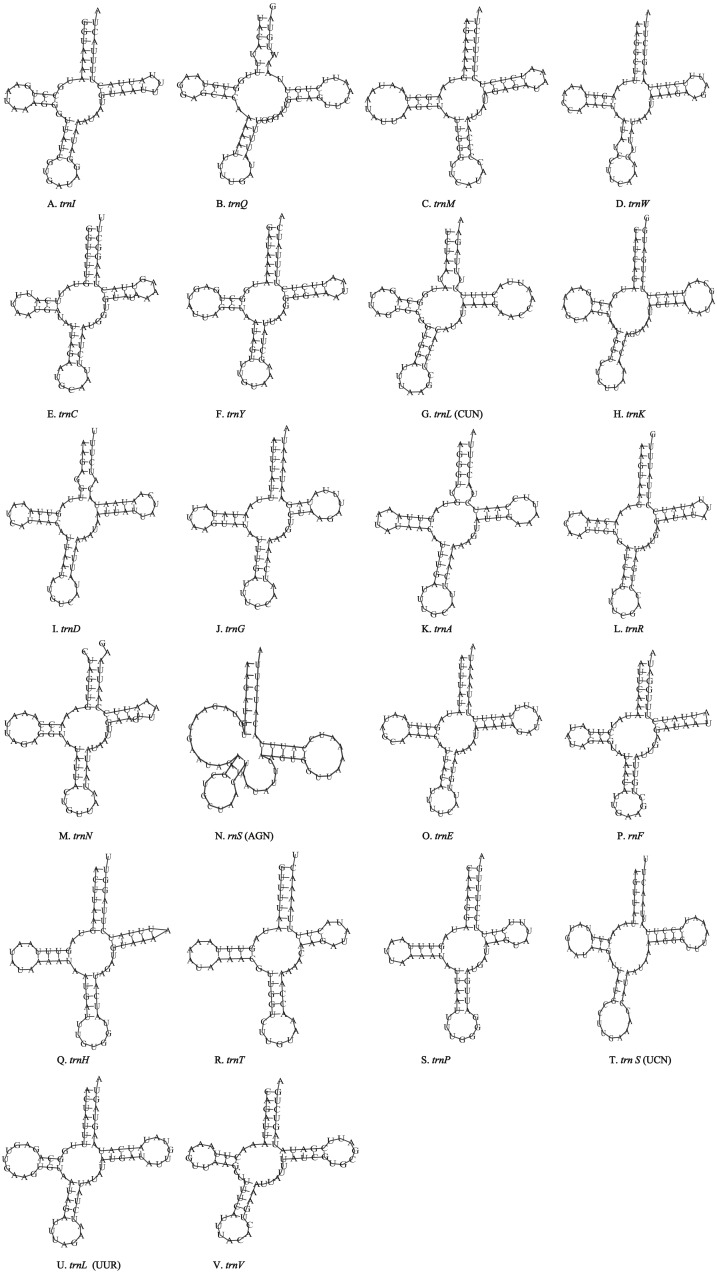
Inferred secondary structures of the 22 tRNA genes of *T. hauseri* mitogenome. (A) *trnI*; (B) *trnQ*; (C) *trnM*; (D) *trnW*; (E) *trnC*; (F) *trnY*; (G) *trnL* (CUN); (H) *trnK*; (I) *trnD*; (J) *trnG*; (K) *trnA*; (L) *trnR*; (M) *trnN*; (N) *trnS* (AGN); (O) *trnE*; (P) *trnF*; (Q) *trnH*; (R) *trnT*; (S) *trnP*; (T) *trnS* (UCN); (U) *trnL* (UUR); (V) *trnV*.

Unlike the typical set of 22 tRNA genes in metazoan mitogenomes, there were 26 tRNA genes including an extra two copies of *trnA*-*trnR* predicted in the *P. polyacantha* whereas *T. hauseri* had a typical set of 22 tRNA genes. The secondary clover-leaf structures of tRNA genes identified in the mitogenome of *P. polyacantha* and *T. hauseri* are shown in Figs. 4A–4Z and 5A–5V. The lengths of these tRNA genes varied from 63 bp to 72 bp. All the predicted tRNAs displayed the typical clover-leaf secondary structure, except for *trnS* (AGN), where the DHU arm appears to be replaced by unpaired nucleotides, a feature typical of other animal mitochondria (Wolstenholme, 1992). All the mismatched base pairs found were U-G pairs, and there were 24 and 25 mismatched base pairs in the *P. polyacantha* and *T. hauseri* sequences, respectively. In addition, unmatched U–U base pairs were observed in both two mitogenomes.

The mitogenome of *P. polyacantha* had an extra 2 copies of *trnA-trnR* and formed the gene cluster *ND3-trnA-trnR-trnA-trnR-trnA-trnR-trnN-trnS-trnE-trnF-ND5.* Three *trnA* genes were identical whereas the first *trnR* was a little different from the other two because it had an extra 2 bp nucleotide “TA” on the TψC arm (Figs. 4J–4O). Duplication of tRNA is a common phenomenon in mantis mitogenomes. Ye et al. (2016) found that 4 mantises (*Creobroter gemmata*, *Mantis religiosa*, *Statilia* sp., *Theopompa* sp.-HN) had 2–8 copies of *trnR*. In addition, *Statilia* sp. had 5 *trnW2* copies as well as 6 *trnR* forming the gene cluster *ND3-trnA-trnR-trnR-trnW2-trnR-trnR-trnW2-trnW2-trnR-trnW2-trnN-trnS-trnE-trnF-ND5*. Zhang et al. (2018a) found that the mitogenomes of *S. porioni* and *Schizocephala bicornis* had 3 identical *trnK* and 5 identical *trnI*, respectively. Evidence for tRNA duplication has also been found in other insect orders such as Ephemeroptera (Zhang et al., 2008), Hymenoptera (Cameron et al., 2008) and Lepidoptera (Hu et al., 2010). However, the occurrence of three copies of *trnA-trnR* in *P. polyacantha* is the first such report in Insecta although in the mitogenome of *Brontostoma colossus* (Kocher et al., 2014), *trnA-trnR* may have been duplicated at least once. This is because the gene cluster *ND3-trnR-trnA-trnR-ND5* was found in *B. colossus* and there were 40 bp intergenic nucleotides between *ND3* and *trnR*, 20 bp of which showed high similarity to *trnA* (100%).

### A+T-rich region and intergenic regions

The A+T-rich region of the insect mitogenome is equivalent to the control region of vertebrate mitogenomes and harbors the origin sites for transcription and replication (Andrews et al., 1999; Yukuhiro et al., 2002; Cameron, 2014a; Du et al., 2016). The A+T-rich regions of *P. polyacantha* and *T. hauseri* mitogenomes were located between *12S rRNA* and *trnI* with lengths of 709 bp and 687 bp, respectively (Figs. 1A–1B), which is similar to *S. porioni* (685 bp). The length of the A+T-rich region is variable, generally ranging from 639 bp in *Mantis religiosa* to 1,775 bp in *Theopompa* sp.-HN (Ye et al., 2016). In the mitogenomes of *P. polyacantha* and *T. hauseri*, the contents of A+T were 77.39% and 76.34%, respectively, similar to *S. porioni* (76.39%). The A +T-rich regions of *P. polyacantha* and *S. porioni* genomes both showed positive AT-skew values whereas *T. hauseri* showed a negative skew. All A+T-rich regions of the three Toxoderidae species displayed negative GC-skew values. Unlike the species *Anaxarcha zhengi*, *Hierodula formosana*, *Rhombodera valida*, *Tamolanica tamolana*, *Theopompa* sp.-YN and *Theopompa* sp.-HN that showed different copies of tandem repetitive sequences (Cameron et al., 2008; Ye et al., 2016; Zhang & Ye, 2017), we failed to find any tandem repetitive sequences in *P. polyacantha* and *T. hauseri* using Tandem Repeat Finder V 4.07 (http://tandem.bu.edu/trf/trf.html) (Benson, 1999).

### Intergenic regions

**Figure 6:**
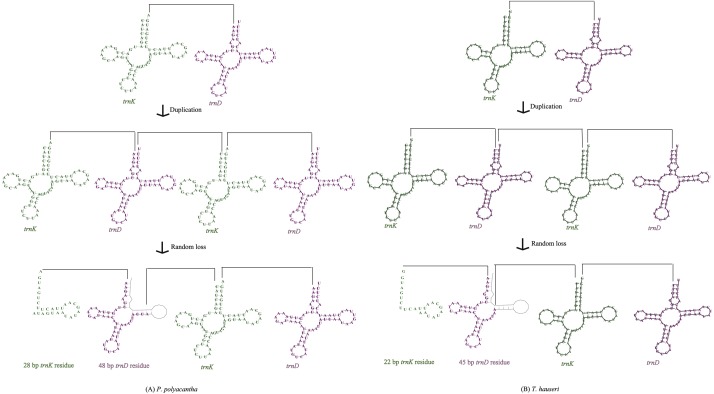
Proposed mechanism of gene arrangements in *P. polyacantha* (A) and *T. hauseri* (B).

The mitogenomes of most insect species seem to be economical (Boore, 1999) although large intergenic regions exist in some species. For example, large intergenic regions located between *trnM* and *ND2* were observed in seven Paramantini species with variable lengths ranging from 296 bp in *Tamolanica tamolana* to 1,541 bp in *Hierodula patellifera* (Cameron, Barker & Whiting, 2006; Tian et al., 2017; Zhang & Ye, 2017; Zhang et al., 2018a). In the mitogenome of *P. polyacantha*, there was a total of 305 bp of intergenic space between genes, of which there were 10 locations of intergenic lengths smaller than 8 bp, four locations of intergenic lengths between 10 bp and 20 bp and five locations of intergenic lengths longer than 20 bp (Table S1). The longest intergenic region was located between *COX2* and *trnK* (76 bp), 28 bp of which showed high similarity to *trnK* (100%) whereas the other 48 bp showed high similarity to *trnD* (100%). This gene arrangement can be explained by the tandem duplication/random loss mode (TDRL) (Fig. 6A). Firstly, the region of *trnK*-*trnD* was tandem duplicated once. Secondly, the random deletion of a portion of one of the *trnK*-*trnD* pairs occurred to form a 76 bp partial “*trnK*-*trnD*” residue. The mitogenome of *T. hauseri* contained a total of 210 bp of intergenic space spread over 21 regions with sizes ranging from 1 to 67 bp (Table S2). The longest intergenic region was located between *COX2* and *trnK* (67 bp) and can also be explained by TDRL mode (Fig. 6B). Accordingly, 22 bp were similar to *trnK* (100%) and the remaining 45 bp were similar to *trnD* (100%). Thus, we can infer that *trnK-trnD* was duplicated at least once and then randomly deleted. This feature was also found in the mitogenome of *S. porioni*, suggesting that it could be synapomorphic for Toxoderidae, because this characteristic has only been found in Toxoderidae whereas the other families of Mantodea have only 0–2 nucleotides located between *COX2* and *trnK*. In addition to the intergenic region between *COX2* and *trnK*, the mitogenome of *S. porioni* had another two *trnK* genes and 2 *trnD* residues forming the arrangement *COII*-*trnK* *-*trnD* *-trn*K*-*trnD* *-trn*K*-*trnD* *-trn*K*-*trnD-ATP8* (*trnK* * and *trnD* * represent tRNA residues) (Zhang et al., 2018a).

**Figure 7:**
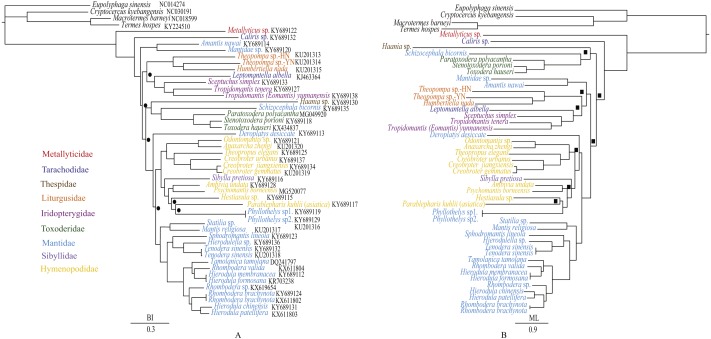
Phylogenetic relationships of Mantodea inferred from BI analysis (A) and ML analysis (B). Black circles at nodes of the BI analysis indicate PP < 0.95 whereas black squares at nodes of the ML analysis indicate BP < 75 (B).

### Phylogenetic analyses

The phylogenetic relationships inferred from BI analysis (Fig. 7A) and ML analysis (Fig. 7B) had somewhat different topologies, the BI topology being almost identical to that of Zhang et al. (2018a). In BI analysis, the main topology was as follows: (*Amantis nawai* (Mantidae) + Mantidae sp.) + ((Liturgusidae + (*Leptomantella albella* + Iridopterygidae)) + ((*Haania* sp. + (*Schizocephala bicornis* + Toxoderidae)) + remaining mantises)). However, in ML analysis, the main topology was (*Haania* sp. + (*Schizocephala bicornis* + Toxoderidae)) + (((*Amantis nawai* + Mantidae sp.) + (Liturgusidae + (*Leptomantella albella +* Iridopterygidae))) + remaining mantises). The difference was mainly caused by the unstable position of three clades: (*Amantis nawai* + Mantidae sp.), (Liturgusidae + (*Leptomantella albella* + Iridopterygidae)) and (*Haania* sp. + (*Schizocephala bicornis* + Toxoderidae)). We included three Toxoderidae taxa (*P. polyacantha*, *S. porioni* and *T. hauseri*) and they formed a monophyletic clade which was also the sister clade to *Schizocephala bicornis* (Mantidae). Toxoderidae as the sister clade to *Schizocephala bicornis* was also found in Zhang et al. (2018a) but was reported to be the sister clade to Oxyothespinae (Mantidae) in Svenson & Whiting (2009). The species of Oxyothespinae (Mantidae) was not including in this study. To address this discrepancy, future studies should increase the number of species used, especially with samples of Oxyothespinae, Tarachodinae and Amelinae.

## Conclusion

We successfully determined the complete mitogenomes of *P. polyacantha* and *T. hauseri* and the two mitogenomes showed similar gene characteristics to other mantis mitogenomes. An extra two copies of *trnA-trnR* was found in the mitogenome of *P. polyacantha*, which was the first report of this in an insect mitogenome, and may give us a useful model for studying the mechanisms of the tRNA duplications. The presence of *trnK*-*trnD* residues between *COX2* and *trnK* could be synapomorphic for Toxoderidae and can be explained by the tandem duplication/random loss model (TDRL). Both BI analysis and ML analysis showed that Toxoderidae was monophyletic and that *P. polyacantha* was a sister clade to *T. hauseri* and *S. porioni*.

##  Supplemental Information

10.7717/peerj.4595/supp-1 Supplemental Information 1 The mt genome of Toxodera hauseri in GenBank format

10.7717/peerj.4595/supp-2 Supplemental Information 2 The mt genome of Paratoxodera polyacantha in GenBank format

10.7717/peerj.4595/supp-3 Table S1 Location of features in the mtDNA of* P. polyacantha*

10.7717/peerj.4595/supp-4 Table S2 Location of features in the mtDNA of *T. hauseri*

## Additional Information and Declarations

### Data Availability

The following information was supplied regarding data availability: *P. polyacantha* (MG049920) and *T. hauseri* (KX434837) in the GenBank database.
