# Supplementary material for: Gene characteristics of the complete mitochondrial genomes of Paratoxodera polyacantha and Toxodera hauseri (Mantodea: Toxoderidae)
Source: PeerJ. 2018 Apr 19;6:e4595. doi: 10.7717/peerj.4595 (PMC5911385; doi:10.7717/peerj.4595)
Supplement: Table S1 [file peerj-06-4595-s003.docx]

**Table S1** Location of features in the mtDNA of *P. polyacantha*

| **Gene** | **Strand** | **Position** | **Length**  **(nuc.)** | **Anticodon** | **Start**  **codon** | **Stop**  **codon** | **Intergenic**  **nucleotides** |
| --- | --- | --- | --- | --- | --- | --- | --- |
| *trnI* | + | 1-66 | 66 | GAT |  |  | +23 |
| *trnQ* | - | 90-159 | 70 | TTG |  |  | +2 |
| *trnM* | + | 162-229 | 68 | CAT |  |  | 0 |
| *ND2* | + | 230-1261 | 1032 |  | ATG | TAA | +2 |
| *trnW* | + | 1264-1332 | 69 | TCA |  |  | -1 |
| *trnC* | - | 1332-1394 | 63 | GCA |  |  | 0 |
| *trnY* | - | 1395-1459 | 65 | GTA |  |  | 0 |
| *COX1* | + | 1460-3016 | 1557 |  | ATA | TAA | +26 |
| *trnL* (CUN) | + | 3043-3114 | 72 | TAA |  |  | +4 |
| *COX2* | + | 3119-3802 | 684 |  | ATG | TAG | +77 |
| *trnK* | + | 3880-3951 | 72 | CTT |  |  | +1 |
| *trnD* | + | 3953-4022 | 70 | GTC |  |  | 0 |
| *ATP8* | + | 4023-4181 | 159 |  | ATT | TAA | -7 |
| *ATP6* | + | 4175-4855 | 681 |  | ATG | TAA | -1 |
| *COX3* | + | 4855-5641 | 787 |  | ATG | T | 0 |
| *trnG* | + | 5642-5705 | 64 | TCC |  |  | 0 |
| *ND3* | + | 5706-6059 | 354 |  | ATG | TAA | +5 |
| *trnA* | + | 6065-6129 | 65 | TGC |  |  | +4 |
| *trnR* | + | 6134-6203 | 70 | TCG |  |  | +45 |
| *trnA* | + | 6249-6313 | 65 | TGC |  |  | +4 |
| *trnR* | + | 6318-6385 | 68 | TCG |  |  | +45 |
| *trnA* | + | 6431-6495 | 65 | TGC |  |  | +4 |
| *trnR* | + | 6500-6567 | 68 | TCG |  |  | 0 |
| *trnN* | + | 6568-6632 | 65 | GTT |  |  | 0 |
| *trnS* (AGN) | + | 6633-6699 | 67 | GCT |  |  | 0 |
| *trnE* | + | 6700-6769 | 70 | TTC |  |  | -5 |
| *trnF* | - | 6773-6836 | 64 | GAA |  |  | 0 |
| *ND5* | - | 6837-8556 | 1720 |  | ATA | T | +12 |
| *trnH* | - | 8569-8631 | 63 | GTG |  |  | +8 |
| *ND4* | - | 8640-9971 | 1332 |  | ATG | TAA | -7 |
| *ND4L* | - | 9965-10246 | 282 |  | ATG | TAA | +11 |
| *trnT* | + | 10258-10320 | 63 | TGT |  |  | 0 |
| *trnP* | - | 10321-10383 | 63 | TGG |  |  | -7 |
| *ND6* | + | 10377-10880 | 504 |  | ATT | TAA | -1 |
| *CYTB* | + | 10880-12016 | 1137 |  | ATG | TAA | +12 |
| *trnS* (UCN) | + | 12029-12102 | 74 | TGA |  |  | +8 |
| *ND1* | - | 12111-13046 | 936 |  | ATG | TAA | +10 |
| *trnL* (UUR) | - | 13057-13126 | 70 | TAG |  |  | 0 |
| *16S rRNA* | - | 13127-14514 | 1388 |  |  |  | 0 |
| *trnV* | - | 14446-14515 | 70 | TAC |  |  | 0 |
| *12S rRNA* | - | 14447-15289 | 843 |  |  |  | 0 |
| A+T-rich region |  | 15290-15999 | 710 |  |  |  |  |
