# Supplementary material for: Gene characteristics of the complete mitochondrial genomes of Paratoxodera polyacantha and Toxodera hauseri (Mantodea: Toxoderidae)
Source: PeerJ. 2018 Apr 19;6:e4595. doi: 10.7717/peerj.4595 (PMC5911385; doi:10.7717/peerj.4595)
Supplement: Table S2 [file peerj-06-4595-s004.docx]

**Table S2** Location of features in the mtDNA of *T. hauseri*

| **Gene** | **Strand** | **Position** | **Length**  **(nuc.)** | **Anticodon** | **Start**  **codon** | **Stop**  **codon** | **Intergenic**  **nucleotides** |
| --- | --- | --- | --- | --- | --- | --- | --- |
| *trnI* | + | 1-65 | 65 | GAT |  |  | +24 |
| *trnQ* | - | 90-159 | 70 | TTG |  |  | +2 |
| *trnM* | + | 162-232 | 71 | CAT |  |  | 0 |
| *ND2* | + | 233-1264 | 1032 |  | ATG | TAA | -1 |
| *trnW* | + | 1263-1330 | 68 | TCA |  |  | +7 |
| *trnC* | - | 1338--1402 | 65 | GCA |  |  | +1 |
| *trnY* | - | 1404-1470 | 67 | GTA |  |  | +24 |
| *COX1* | + | 1495-3030 | 1536 |  | TTG | TAA | +4 |
| *trnL* (CUN) | + | 3035-3102 | 68 | TAA |  |  | +3 |
| *COX2* | + | 3106-3789 | 684 |  | ATG | TAA | +67 |
| *trnK* | + | 3857-3928 | 72 | CTT |  |  | +1 |
| *trnD* | + | 3930-3996 | 67 | GTC |  |  | 0 |
| *ATP8* | + | 3997-4155 | 159 |  | ATC | TAA | -4 |
| *ATP6* | + | 4152-4829 | 678 |  | ATA | TAA | -1 |
| *COX3* | + | 4829-5616 | 788 |  | ATG | T | -1 |
| *trnG* | + | 5616-5680 | 65 | TCC |  |  | 0 |
| *ND3* | + | 5681-6034 | 354 |  | ATG | TAA | +5 |
| *trnA* | + | 6040-6104 | 65 | TGC |  |  | +4 |
| *trnR* | + | 6109-6175 | 67 | TCG |  |  | +18 |
| *trnN* | + | 6194-6259 | 66 | GTT |  |  | +1 |
| *trnS* (AGN) | + | 6261-6325 | 65 | GCT |  |  | +1 |
| *trnE* | + | 6327-6397 | 71 | TTC |  |  | +4 |
| *trnF* | - | 6402-6465 | 64 | GAA |  |  | 0 |
| *ND5* | - | 6466-8191 | 1726 |  | ATG | T | 0 |
| *trnH* | - | 8192-8255 | 64 | GTG |  |  | +7 |
| *ND4* | - | 8263-9600 | 1338 |  | ATG | TAA | -7 |
| *ND4L* | - | 9594-9875 | 282 |  | ATG | TAA | +2 |
| *trnT* | + | 9878-9941 | 64 | ATG |  |  | 0 |
| *trnP* | - | 9942-10004 | 63 | TGG |  |  | +2 |
| *ND6* | + | 10007-10510 | 504 |  | ATC | TAA | -1 |
| *CYTB* | + | 10510-11646 | 1137 |  | ATG | TAA | +13 |
| *trnS* (UCN) | + | 11660-11729 | 70 | TGA |  |  | +18 |
| *ND1* | - | 11748-12677 | 930 |  | ATA | TAA | +2 |
| *trnL* (UUR) | - | 12680-12750 | 71 | TAG |  |  | 0 |
| *16S rRNA* | - | 12751-14072 | 1322 |  |  |  | 0 |
| *trnV* | - | 14073-14142 | 70 | TAC |  |  | 0 |
| *12S rRNA* | - | 14143-14929 | 787 |  |  |  | 0 |
| A+T-rich region |  | 14930-15616 | 687 |  |  |  |  |
